# High-Energy Density Pure Polyvinylidene Difluoride with the Magnetic Field Modulation of Free-Volume Pore Size and Other Microstructures

**DOI:** 10.3390/polym16212979

**Published:** 2024-10-24

**Authors:** Zhaoting Liu, Jiale Qiao, Chao Liu, Shuotong Qiao

**Affiliations:** 1 School of Physics and Electronic Engineering, Northeast Petroleum University, No. 199, Fazhan Road, Daqing 163318, China; liuzhaoting@nepu.edu.cn (Z.L.); msm-liu@126.com (C.L.); dr_xiaoqiao@163.com (S.Q.); 2 School of Electrical Engineering, Suihua University, No. 18, Huanghe South Road, Suihua 163318, China

**Keywords:** PVDF, positron annihilation, high-energy density

## Abstract

PVDF polymer dielectrics, renowned for their ultra-high-power density, ultra-fast response times, remarkable toughness, and lightweight properties, constitute the essential material foundation for the development of dielectric capacitors. Nevertheless, the low-energy density of these dielectrics presents a challenge to the advancement of dielectric capacitors. In this paper, in the process of preparing monolayer pure PVDF dielectric films by the solution casting method, a fixed-direction magnetic field and a rotating magnetic field were introduced in the horizontal direction, respectively, and this investigation explores the impact of magnetic field modulation on the polymer films’ free-volume pore size, grain size, phase structure, dielectric properties, and energy storage capabilities by altering the duration and orientation of the magnetic field’s influence. This study also discusses how microscopic alterations, particularly in the free-volume pore size, affect the macroscopic dielectric properties. Polymer films treated with a magnetic field of constant orientation for 3 min were obtained with the smallest free-volume hole size of 2.91 Å, the highest γ-phase contents of 54.8%, the smallest grain size of 68 Å, the largest electrical displacement of 10.64 and a very high discharge energy density of 12.68 J/cm^3^ (a 200% enhancement over pure PVDF).

## 1. Introduction

Advanced energy storage technologies are urgently needed to fulfil the goals of the global sustainable energy development strategy [1]. Energy storage technologies are mainly classified into physical energy storage and chemical energy storage. Chemical energy storage, represented by supercapacitors and lithium battery storage, first converts electrical energy into chemical energy, stores it, and eventually releases it in the form of electrical energy for consumer use. It is characterised by a long response time. Physical energy storage represented by dielectric capacitors is based on the conversion mechanism of electric dipoles to store charge and electrostatic energy [2]. Characterised by ultrahigh power density (megawatt level), ultra-fast response times (microsecond level), low loss, a long cycle life (t > 10^5^ h), and high operating voltage, dielectric capacitors are widely used in new energy vehicles, AC and DC converters, power inverters, aerospace, pulsed military weaponry, and electronic medical devices [3].

The performance of dielectric capacitors is mainly determined by the performance of the dielectric material. Compared with ceramics, polymer-based dielectric materials have the advantages of being lightweight, having easy preparation, a low price, higher breakdown voltage, etc. They are an important material basis for the future integration and miniaturisation of power electronic devices, the popularisation of smart, flexible wearable electronic devices, the scientific and technological innovation of the new energy automobile industry, and the development of energy-saving and environmentally friendly new energy technologies [4].

This was demonstrated in a number of studies where the dielectric constant of flexible polymer PVDF is usually higher than that of general polymers, with a high energy density of 6.23 J/cm^3^ (commercial BOOP 2.2 J/cm^3^) and good thermal stability, UV resistance, and chemical resistance to most chemicals and solvents, which has a high application value in the fields of power electronics, aerospace, new energy vehicles, etc., and is one of the alternative high-quality materials for dielectric energy storage capacitors [5].

PVDF is a semi-crystalline polymer with five crystalline phases reported so far, namely the α-phase with the transintertwisted conformation TGTG, where the phases contain dipoles aligned in opposite directions, which generate forces that cancel each other out and lead to non-polarity. The β-phase of the all-trans planar sawtooth conformation TTTT with dipoles perpendicular to the axial chain and oriented in the same direction allows the formation of ferroelectric domains and generates the highest spontaneous polarisation. For the γ phase of the triple-trans conformational and intertwisted TTTG, the polarisation is intermediate between the α and β phases because the partitioning of the dipole-generating force along the axial direction does not contribute to the polarisation. The δ phase is the polar version of the α phase, and ε is a total of five crystalline phases, which are formed under different conditions and which can be transformed into each other under certain conditions [6]. The dipole polarity of monomeric vinylidene fluoride can reach 5 × 10^−30^–8 × 10^−30^ C·m [7]. Among the three common crystalline phases of PVDF-based polymers, while the β-phase has more prominent piezoelectric properties and excellent dielectric properties in much of the literature, the α-phase has a lower residual polarisation than the β-phase, which is also conducive to the composites exhibiting superior charge/discharge efficiencies and discharge energy densities [8,9]. The quenched γ phase can withstand a higher external electric field strength, lower residual polarisation, and higher discharge energy density [10,11].

The typical structure of a dielectric capacitor is a simple sandwich structure with metal-conducting electrodes plated on both sides of the dielectric material. The principle of charge and discharge is that when an electric field is applied to both sides of the electrodes, the dipole inside the dielectric is polarised and electrostatic energy is stored in the polarised dipole. When a circuit is applied to the dielectric capacitor, the polarised dipole starts to depolarise, releasing electrostatic energy corresponding to the discharge process. However, the polarised dipole cannot be used to discharge completely, and often, residual polarisation is produced during the discharge process due to thermal losses. The smaller the residual polarisation, the greater the capacitor’s discharge energy density and, consequently, the higher the efficiency of the dielectric energy storage capacitor. The discharge energy density (*U_discharge_*) can be calculated by the following Equation (1):

(1) $$U_{discharge}=\int_{D_{\min}}^{D_{\max}}EdD,$$

*D* = *ε*_0_*ε_r_E* = *ε*_0_*E* + *P* (2)

where *D* represents the electric displacement, *E* denotes the breakdown strength, and both D and polarisation (P) are related to Equation (2); ε_0_ signifies the dielectric constant in a vacuum, ε_r_ is the real part of the relative dielectric constant, and *P* stands for polarisation.

For linear polymer dielectrics, as indicated by Equation (3), the discharge energy density is proportional to the real part of the material’s dielectric constant and the square of the breakdown strength.

(3) $$U_{Discharge}=\frac{1}{2}\varepsilon_{0}\varepsilon_{r}E_{b}^{2},$$

Considerable research efforts have been devoted to the energy storage performance of polymer dielectric materials, mainly in the following areas: all-organic polymers [12,13], organic polymers modified with inorganic fillers (ceramics, conductive materials, semiconducting materials, and core–shell fillers) of different dimensions (zero-dimensional, one-dimensional, and two-dimensional) [14,15,16,17,18], chain structure design [19] externally applied field treatments [20], and multilayers or gradient design. Much progress has been made in this field, but cumbersome preparation procedures, high costs, an inability to mass produce, and the nanoaggregation of van der Waals forces have constrained the development of dielectric capacitors. Therefore, there is an urgent need for simple and fast processes to prepare high-energy-density capacitors.

Recent experiments in this area have suggested that free-volume pore size has a significant effect on polarisation and dielectric properties and that the volume of a polymer is partly the intrinsic volume occupied by the molecular chains and partly the free volume of the polymer, which is formed by the spatial gaps created by the curling and intertwining of many molecular chains [21,22]. The free volume can either provide space for the rotation of the molecular chains to form different phase structures or it can be seen as the second phase of the pure polymer, “air”, which is a defect in the polymer. Small changes in the microcosm can create very different macroscopic properties. However, to the best of our knowledge, no externally applied magnetic field has been performed on pure PVDF polymers to modulate the free volume, phase structure, and grain size or to study the effect on the microstructure and energy storage properties.

The purpose of this paper is to study the effect of magnetic field modulation on the phase structure, free volume, grain size, dielectric properties and energy storage properties of PVDF polymers, as well as the link between microscopic quantities and macroscopic properties by introducing a parallel magnetic field in the plane direction of PVDF polymers, and to ultimately be able to obtain monolayers of pure PVDF with a higher energy density under a simple preparation process using a change in the time and direction of the magnetic field treatment dielectric film.

## 2. Experimental Section

### 2.1. Materials

Polyvinylidene fluoride (PVDF) was purchased from Shanghai Sanfu New Materials Co., Ltd., Shanghai, China. N,N-dimethylformamide (DMF) was purchased from Shanghai Sinopharm Group Chemical Reagent Co., Shanghai, China. Deionised water was self-made, and all chemicals or reagents were analytically pure and not further processed.

### 2.2. Preparation of Polymer Films

**Figure 1:**
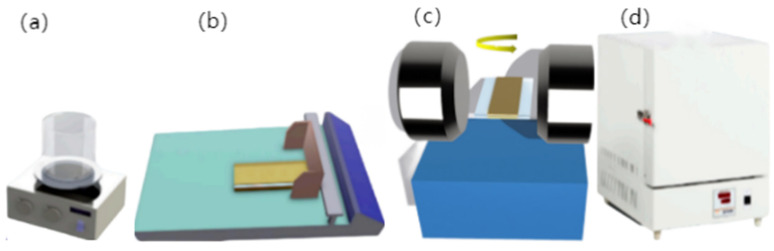
(**a**–**d**) The processes of mixing, smearing, magnetron sputtering, and drying, respectively.

As shown in Figure 1, polymer films were prepared by the solution casting method; firstly, 6 g of PVDF was weighed and added to 60 mL of a DMF solution in small amounts several times and stirred until PVDF was completely dissolved in DMF. After resting for the proper time and slowly pouring on clean glass substrates, the film was coated on the film coating machine to a certain thickness, and the coated film was put into the static/rotating magnetic field for a short time under the applied physical field treatment, respectively, at the fastest speed. Finally, the film was placed in a drying oven at a high temperature of 180 °C for 10 min, during which time an appropriate amount of ice water mixture was prepared by freezing distilled water and placed in a large clean container. The polymer film was removed from the drying oven after high-temperature drying, immediately immersed in the ice water, and later removed and wiped clean before continuing to bake at a low temperature of 80 °C for 6 h to produce the sample we needed. The film without magnetic field treatment was recorded as non, the magnetic field parallel to the film direction and the direction of the invariant according to the processing minutes were recorded as M1, M3, and M5, respectively, parallel to the direction of the film rotating magnetic field (a rotational period of 20 s) according to the processing minutes, which were recorded as RM1, RM3, and RM5, respectively.

### 2.3. Characterisation Techniques

The D8-Advance X-ray diffractometer purchased from Bruker Corporation, Karlsruhe, Germany was used to analyse the physical structure of the films, and CuKα rays with a wavelength of 0.15406 nm were selected at room temperature, with an operating voltage of 40 kV, an operating current of 40 mA, a scanning speed of 5/min, a step size of 0.02 and a scanning range of 10–90°. The functional groups inside the thin film samples were analysed using a JASCO-6100 Fourier-transform, which was purchased from JASCO Corporation, Tokyo, Japan infrared spectroscopy (FTIR) in total reflection (ATR) mode in the wavelength range of 400–4000 cm^−1^. The magnetic field regulation of the polymer films was based on a magnetic field generator, model WD-175, from Yingpu Magnetoelectric Technology Development Co., Ltd., Changchun, China, using a constant magnetic field of 0.8 T. Before the electrical property test, the polymer film was plated with an equal area of metal electrodes on both sides of the polymer film, and a broadband dielectric spectrometer model Alpha-A from Novocontrol, Montabaur, Germany, was used to measure the ambient temperature dielectric properties, variable temperature dielectric properties, and variable temperature modulus curves, in the frequency range of 10^0^ Hz to 10^7^ Hz, with the temperature range of −40 °C to 80 °C. The polymer film was also tested by Radiant Premier II ferroelectric spectrometer, which was published by Redmond, WA, USA. The ferroelectric properties were measured using a Radiant Premier II ferroelectric test system, including D–E hysteresis loops and leakage currents, respectively, at a test frequency of 10 Hz. The discharge energy density and charge/discharge efficiency were calculated from the hysteresis lines.

## 3. Results and Discussion

### 3.1. XRD and Grain Size

**Figure 2:**
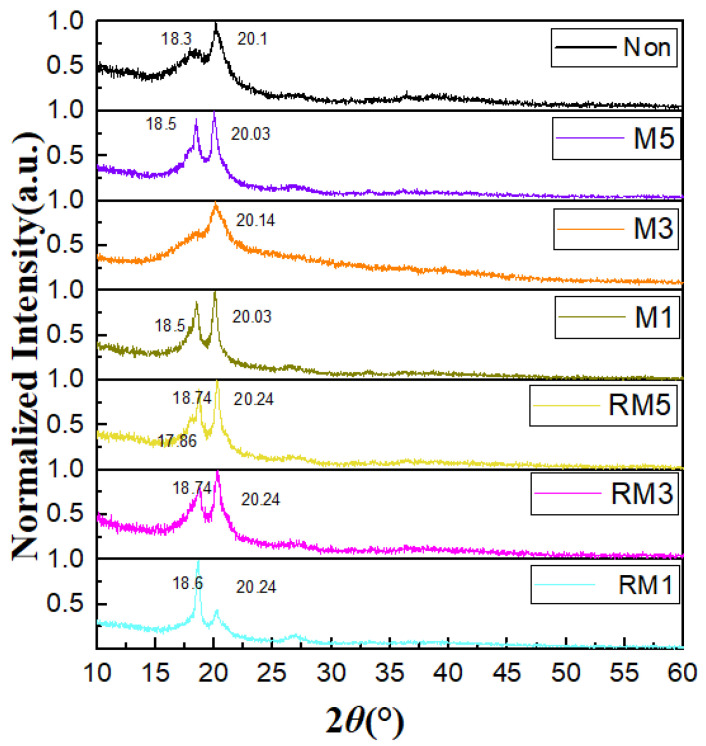
XRD patterns of PVDF.

The diffraction peaks of the films can be observed in Figure 2’s XRD pattern. The diffraction peaks of the PVDF polymer films without magnetic field treatment are 18.3° and 20.10° corresponding to the (020) and (110) crystal planes of the α-phase and γ-phase, respectively. Firstly, the diffraction peaks of PVDF-based polymer films treated with a magnetic field for 1 min and 5 min analysed under horizontal static conditions were very similar at 18.5° and 20.3° corresponding to the (020) and (110) crystal planes of γ phase. The diffraction peaks near 18.5° of the PVDF polymer film treated with a magnetic field for 3 min obviously disappeared, and only the diffraction peak near 20.14° corresponding to the (110) crystal plane of the γ-phase was left, and this film exhibited a typical γ-phase. This indicates that it is possible that the magnetic field inhibits the crystallisation of PVDF along the direction of the perpendicular film. The time of magnetic field action changes the phase structure of PVDF polymers. The following analysis of the horizontal rotation condition with the magnetic field treatment for 1 min includes the strong diffraction peak at 18.57° and the weak diffraction peak at 20.24°, which corresponds to the (020) and (110) crystal faces of the α-phase, respectively [23]. (The enhanced α phase may have a smaller residual polarisation). Magnetic field treatment for 3 min corresponds to the 18.74° medium and strong diffraction peaks and 20.24° strong diffraction peaks, which correspond to the (020) and (110) crystal planes of the α-phase, respectively. Magnetic field treatment for 5 min is significantly better than a 3 min weak diffraction peak of 17.86°, which corresponds to the (100) crystal plane of the α-phase, and this film exhibits a typical α-phase. Altogether, these results indicate that the films contain both α- and γ-phases and that the peak intensities of the crystalline phases and crystallographic surfaces change as the conditions of horizontal magnetic field direction and time vary. The magnetic field modulation affects the crystallographic direction of PVDF. With the PVDF polymers consisting mainly of α- and γ-phases, it is important to discuss the effect of the content of the γ-phase on energy storage properties, which can be calculated according to Equation (4):

(4) $$F(\gamma )=\frac{x_{\gamma }}{x_{\alpha }+x_{\gamma }}=\frac{A_{\gamma }}{(k_{\gamma }/k_{\alpha })A_{\alpha }+A_{\gamma }}=\frac{A_{\gamma }}{1.26A_{\alpha }+A_{\gamma }}$$

Xα and Xγ in Equation (4) denote the mass fractions of the α and γ phases, respectively, Aα and Aγ denote the absorbance of the material at 764 cm^−1^ and 840 cm^−1^, respectively, and Kα (6.1 × 10^4^ cm^2^ mol^−1^) and Kγ (7.7 × 10^4^ cm^2^ mol^−1^) represent the absorption coefficients of the α and γ phases, respectively. By calculating the order from the smallest to the largest modulation time for the non-magnetic field, horizontal static magnetic field and horizontal rotating magnetic field, the γ-phase contents of the seven samples were 3.97%, 47.7%, 54.8%, 12.6%, 44.8%, 42.9%, 44.2%, respectively. Among them, the γ-phase content of M3 was as high as 54.8%.

The crystallographic surface index, half-height width, and grain size of the PVDF polymer films were analysed based on XRD data using Jade 6.5 software. We compared the half-height width FWHM and grain size on the main peak near 2θ equal to 20.1°, corresponding to the plane of γ phase (110). Here, M3 has the smallest grain size of 6.58 nm, and more characteristic peak parameters can be found in Table 1.

### 3.2. FTIR

**Figure 3:**
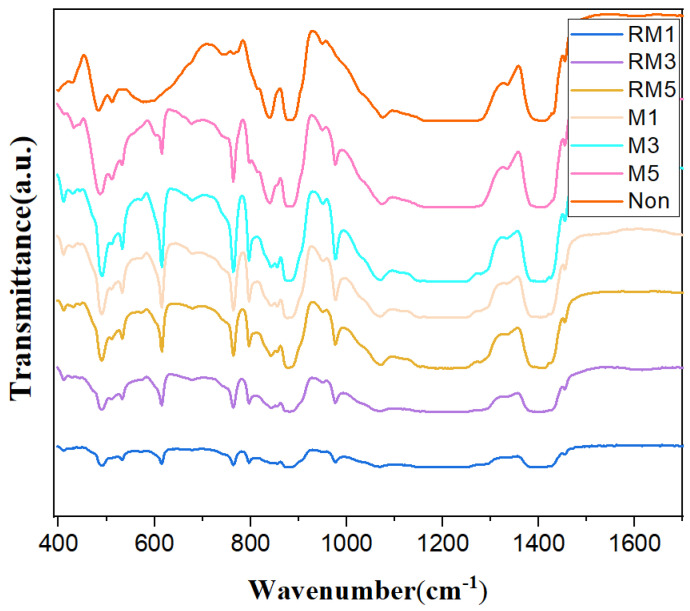
FTIR spectra of pure PVDF.

FTIR spectroscopy has proved to be a useful tool for evaluating different crystal polymorphisms of PVDF. The FTIR spectra of PVDF are shown in Figure 3. The bands around 614 cm^−1^ (*CF*^2^ bending and CCC skeletal vibration), 764 cm^−1^ (*CH*^2^ in-plane or rocking, *CF*^2^ and skeletal bending), 796 cm^−1^ (*CF*^2^ rocking), 976 cm^−1^ (*CH*^2^ rocking, *CF* out-of-plane deformation) [24,25], and 1150 cm^−1^ (*CF*^2^ stretching) are attributed to nonpolar α-phase PVDF. The bands around 482 cm^−1^ (*CF*^2^ bending), 840 cm^−1^ (*CH*^2^ rocking, (*CF*^2^ asymmetrical stretching and skeletal C-C stretching), 883 cm^−1^ (*CF*_2_ rocking), 1401 cm^−1^ (*CH*^2^ scissoring), and 1429 cm^−1^ are attributed to nonpolar γ-phase PVDF [26,27]. The characteristic absorption peak of the –*CH*^2^ group is at 1071 cm^−1^ [28]. The above FTIR mapping results indicate that the main nonpolar α-phase and γ-phase exist in the PVDF matrix, which correspond to the two molecular conformations of ferroelectric polymer PVDF (TGTG’ conformation and T3GT3G’ conformation), respectively, and this result matches with the analysis results of XRD mapping.

### 3.3. Positron Annihilation (PAT)

**Figure 4:**
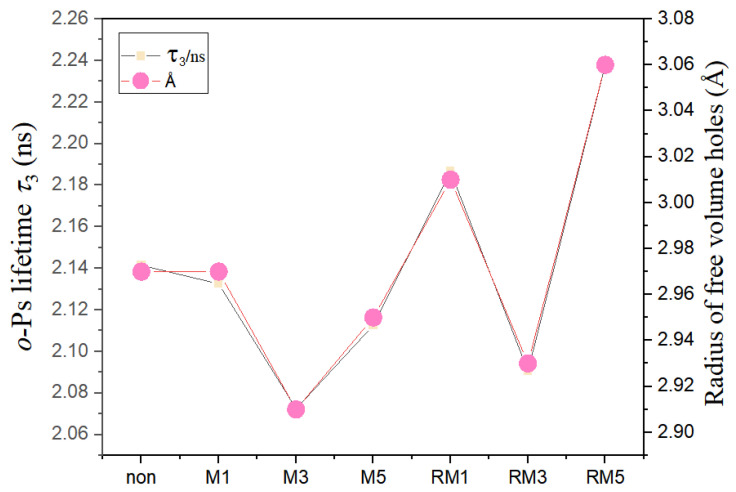
Free-volume pore size of the film.

The effect of magnetic field modulation on the microstructure of polymers, such as free-volume pore size, indirectly changes the electrical properties of polymers. Measuring the free-volume pore size of polymers using positron annihilation lifetime spectroscopy has the advantages of high accuracy and no damage to the original structure. The positron annihilation lifetime spectrum is measured using a conventional fast-fast conformal lifetime spectrometer. A ^22^Na source (with an activity of about 12μCi) was sandwiched between two sheet samples of the same size and thickness (the thickness of the samples was about 1–2 mm), wrapped in tin foil and placed between the two detectors for the measurements, and each spectrum contained 10^6^ counts, which were solved using PATFIT software to obtain the positron lifetime value parameter. All measured spectra were decomposed into three components (τ1, τ2, and τ3) and discretised using PATFIT. The long lifetime component τ3 is attributed to the segregated annihilation of o-Ps in the free-volume holes in the amorphous region, as shown in Figure 4. It can be observed that the o-Ps lifetime τ3 and the free-volume hole size of the field-free modulated films are 2.14 and 2.7 Å, respectively, and the free-volume hole size is almost unchanged after the directionally static magnetic field treatment for 1 min, and the free-volume hole size exhibits an initial decrease. Then, the free-volume pore size shows a tendency to decrease and then increase with the increase in time. Overall, the free-volume pore size was reduced by the static magnetic field. Similarly, the free-volume pore size decreased and then increased with time for films treated with uniformly varying magnetic fields. However, a rotating magnetic field can increase the free-volume pore size.

### 3.4. Dielectric

**Figure 5:**
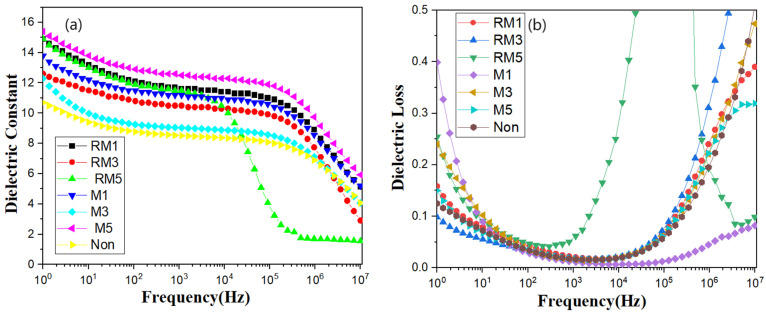
(**a**,**b**) Dielectric constants and dielectric losses of the film, respectively.

Investigating the effect of magnetic field modulation on the dielectric properties of PVDF polymers is extremely significant. In Figure 5a,b, room temperature dielectric curves show that the dielectric constants (dielectric losses) of PVDF films treated with the rational field at 1 kHz are modulated according to the non-magnetic field, the horizontal static magnetic field, and the horizontal rotating magnetic field in the order of time from small to large: 8.51 (0.0166), 11.4 (0.0183), 9.04 (0.0154), 12.5 (0.0173), 11.6 (0.0200), 10.5 (0.0176) and 11.5 (0.0615), respectively. The relative permittivity of the polymer films decreases with increasing frequency over the entire frequency range, with the majority of the sample films stabilising between 10^0^ and 10^5^ Hz, followed by a sharp drop in permittivity and a significant increase in dielectric loss at higher frequencies. As the dipole steering polarisation cannot follow the frequency change in the external electric field, the polarisation inside the composite medium cannot be established in time, so the dielectric constant of the composite medium decreases and the loss increases. The PVDF film without physical field treatment at 1 kHz has a minimum dielectric constant of 8.51, and the horizontal static magnetic field modulation for 5 min has a maximum dielectric constant of 12.5, which is increased by 46.88% compared to no magnetic field. The dielectric constant was optimised after magnetic field conditioning.

Figure 5b shows the loss values of seven films, except for the film treated with a horizontal rotating magnetic field for 5 min, where the loss value at 1 kHz was less than 0.02, the film treated with a horizontal static magnetic field for 1 min had the smallest loss of 0.0111, and the curves of the rest of the films also almost coincided at that point. This indicates that most of the prepared films have low loss, and proper magnetic field modulation can reduce the loss value.

**Figure 6:**
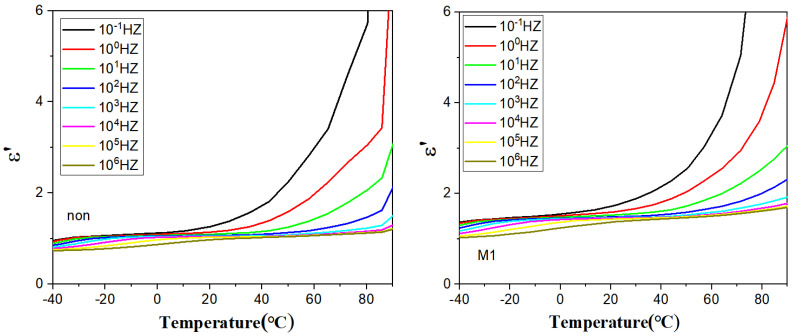

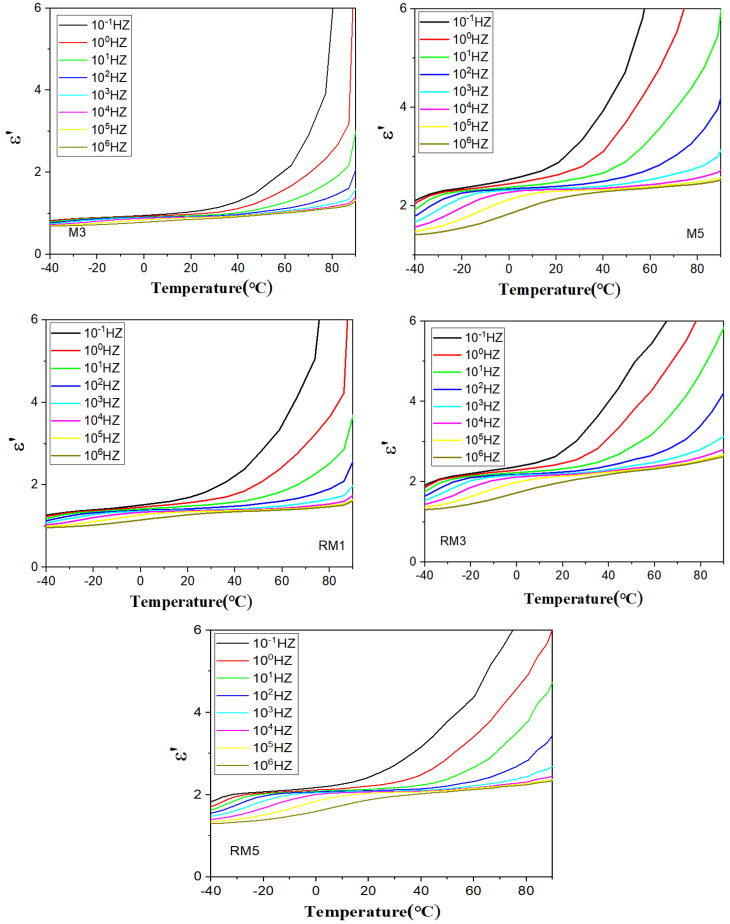
Dielectric modulus imaginary part vs. temperature curve.

Figure 6 shows the dependence of the real part of the dielectric constant on the temperature, with a wave peak in the low-temperature region, which represents the glass transition temperature of PVDF, Tg [29,30,31]. As the frequency increases, the wave peaks move significantly towards the high-temperature region, which typically involves thermally activated relaxation, suggesting that more energy is required to complete the movement of the chain segments at high frequencies.

**Figure 7:**
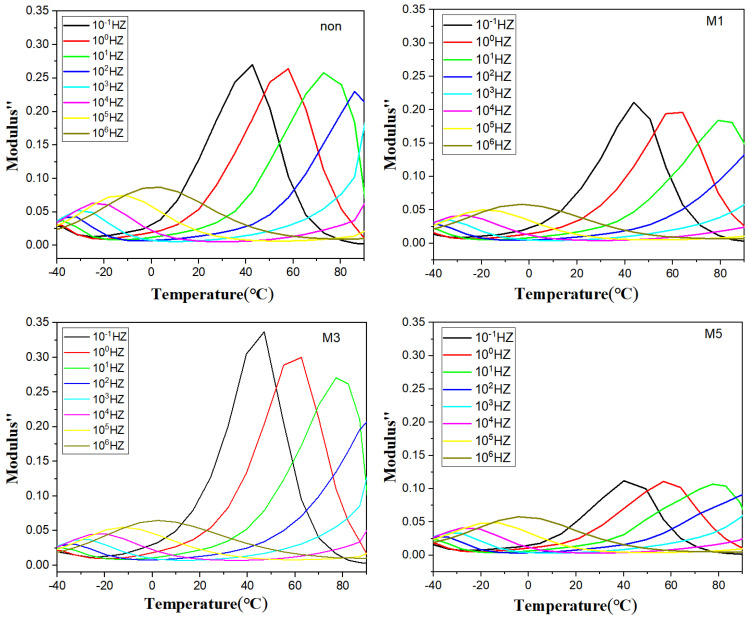

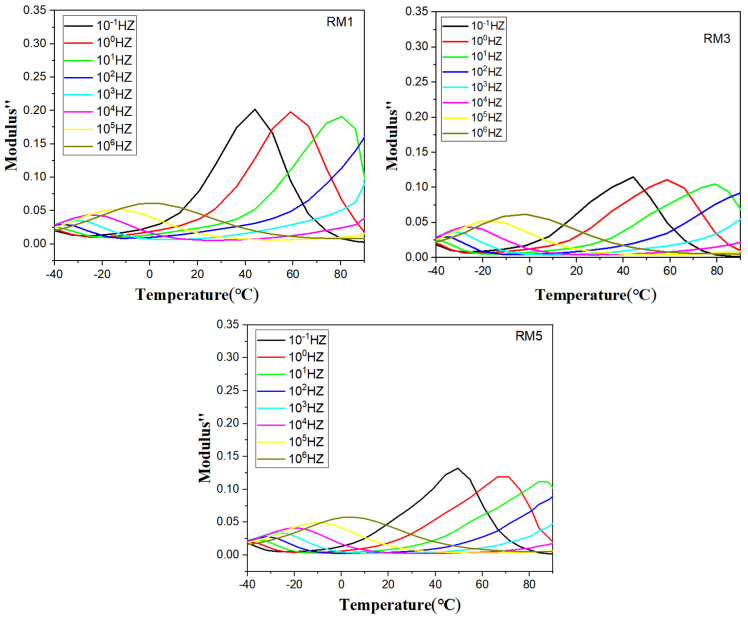
Dielectric modulus imaginary part vs. temperature curve.

Figure 7 shows a plot of the imaginary part of the dielectric modulus of the polymer film versus temperature. As shown in the figure, it is clear that there are two characteristic peaks in the lower and higher temperature regions: the α-relaxation peak in the lower temperature region originates from the relaxation of molecular chains in the amorphous area of the PVDF inside the polymer, and its thermally activated chain movement with increasing temperature leads to a shift in the α-relaxation peak to the higher frequency region. The peaks in the samples gradually move to higher frequencies with increasing temperature, which is due to ionic leaps. M3 has the highest interfacial polarisation peak and may have better energy storage properties.

### 3.5. Leakage Current and Breakdown Strength

The breakdown of PVDF-based polymers is mainly related to the inherent band gap width of the material itself, mechanical deformation, and thermal breakdown from loss. The breakdown strength was calculated using the two-parameter Weibull distribution shown in Equation (5).

(5) $$P(E)=1-\exp{\left(\frac{E}{E_{b}}\right)}^{\beta }$$

where *P* (*E*) denotes the cumulative probability of electrical failure, *E* is the experimental electric field strength at the time of electrical breakdown, *Eb* is the characteristic breakdown strength when the cumulative failure probability is 63.7%, and *β* is the shape of the parameter used to evaluate the degree of dispersion of the breakdown strength, with larger values indicating higher accuracy.

**Figure 8:**
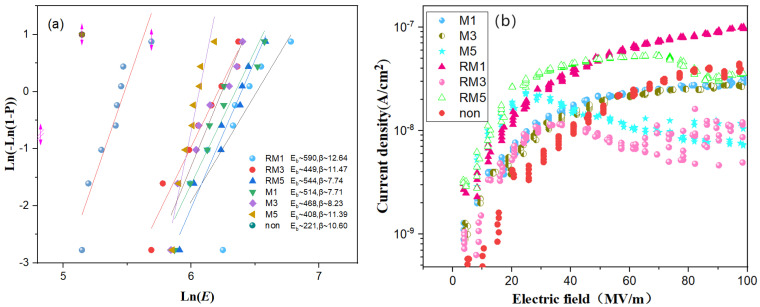
(**a**,**b**) The two-parameter Weibull distribution of breakdown strength and leakage current, respectively.

The PVDF polymer without magnetic field treatment in Figure 8a corresponds to the smallest Weibull distribution of the characteristic breakdown field strength Eb, 221 kV/mm. The rotating magnetic field in the horizontal direction magnetises the PVDF polymer for 1 min with a maximum breakdown strength of 590 kV/mm and *β* of 12.64. The combination of XRD and FTIR suggests that the reason for this may be that the magnetic field alters the crystalline region of PVDF, which changes the molecular structure of PVDF, and the strong α diffraction peaks combined with the weak γ diffraction peaks may enhance the voltage resistance of PVDF polymers.

The leakage current density induced by the electric field can reflect, to some extent, the insulating properties and the internal carrier transport behaviour of the polymer film dielectric material. When an external electric field is applied, leakage currents are generated inside the dielectric due to the formation of conductive pathways. For polymers with high-insulating properties, the leakage current mainly originates from the intrinsic carrier transport in the PVDF volume, e.g., space charge, etc. Thus, the leakage current–electric field curves of the polymers are given in Figure 8b, and the overall leakage current density is roughly in the range of 6.16 × 10^−10^ to 9.8 × 10^−8^ A/cm^2^ in the electric field range of 0–100 kV/mm. At an electric field of 100 kV/mm, the leakage current density of the PVDF decreases from 4.12 × 10^−8^ A/cm^2^ to 4.94 × 10^−9^ A/cm^2^. The leakage current increases with the increase in voltage; the reasons for analysing the above phenomenon are mainly because of the following: a high electric field is caused by the migration of more intrinsic free electrons (carriers) inside the medium, in addition to the high voltage, which promotes the electrode to inject a higher number of mobile-free electrons. When the applied electric field is greater than the dielectric material tolerance limit, intrinsic free electrons and injected free electrons break through the boundaries of the formation of through-currents inside the dielectric. This leads to an increase in the leakage current density inside the polymer dielectric material; the decrease in leakage current indicates, to some extent, that the conductive paths inside the dielectric are not easy to form or are formed in smaller quantities, so the conductive loss is reduced, and the ability to resist breakdown is improved.

### 3.6. Energy Storage Performance

**Figure 9:**
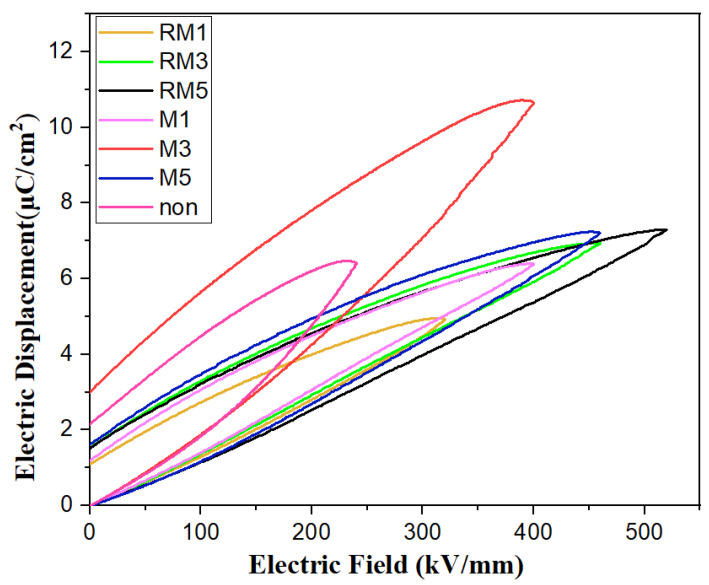
D–E hysteresis loop.

In Figure 9, the maximum potential shifts of PVDF polymer films under 240 kV/mm, 400 kV/mm, 400 kV/mm, 460 kV/mm, 320 kV/mm, 460 kV/mm, and 520 kV/mm electric fields are shown. In D–E, the hysteresis loops are 6.43 μC/cm^2^, 6.38 μC/cm^2^, 10.64 μC/cm^2^, 7.21 μC/cm^2^, 4.93 μC/cm^2^, 6.99 μC/cm^2^, and 7.29 μC/cm^2^, respectively. The film treated with a static horizontal magnetic field for three minutes has the maximum potential shift of 10.64 μC/cm^2^, but the residual polarisation value is higher at about 3.03 μC/cm^2^, and in combination with the XRD plot, the samples are single peaked γ phase, which may have resulted in having excellent maximum polarisation and large residual polarisation. It indicates that the magnetic field modulation favours the dielectric polarisation and reduces residual polarisation, which can effectively prevent the Joule heat generated by the residual polarisation from leading to the thermal breakdown of the film during the charging and discharging process. It coincides with the breakdown performance curve.

**Figure 10:**
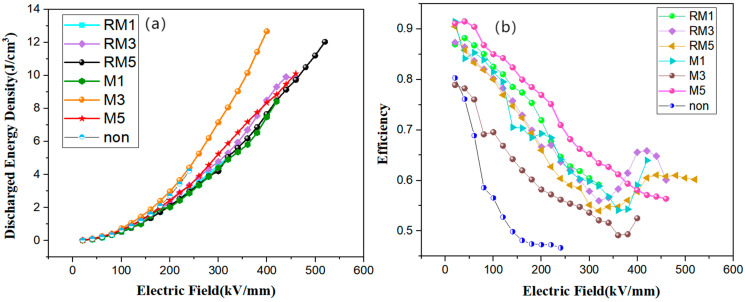
(**a**,**b**) The discharge energy density and charge/discharge efficiency, respectively.

Figure 10 illustrates the discharge energy density (efficiency) values of 4.23118 J/cm^3^ (0.46691), 8.425 J/cm^3^ (0.63977), 12.68185 J/cm^3^ (0.52524), 10.11482 J/cm^3^ (0.56389), 5.03764 J/cm^3^ (0.59 159), 9.83199 J/cm^3^ (0.60142), and 12.03719 J/cm^3^ (0.6019). The maximum discharge energy density of the polymer film treated with a magnetic field of constant direction for 3 min is related to the fact that it has the smallest free-volume pore size of 2.91 Å, the highest γ-phase content of 54.8%, and the smallest grain size of 68 Å, and is not entirely dependent on the decoupling modulation of the dielectric properties. Because the dielectric constant and breakdown strength of some samples are greater than M3, the discharge energy density is less than M3, which is related to a combination of factors such as their free-volume hole size and γ-phase content.

## 4. Conclusions

In this experiment, the effects of directional static and rotating magnetic fields on microstructural and macroscopic electrical properties, such as phase structure, free-volume pore size, grain size, etc., of pure PVDF were investigated in the dielectric wet film formation process. XRD indicates that magnetic field regulation changes the crystalline orientation, phase, and peak intensity of the crystal planes of PVDF. The impact of the γ-phase on discharge energy density was substantiated by quantifying its content, with findings from Fourier-transform infrared spectroscopy (FTIR) corroborating those from X-ray diffraction (XRD). The free-volume holes within the material were assessed using positron annihilation lifetime spectroscopy. Following exposure to a stationary magnetic field for one minute, the dimensions of these holes remained largely constant; however, over time, they exhibited a fluctuation, initially decreasing before increasing. In summary, the stationary magnetic field tended to reduce the size of the free-volume holes. A similar pattern was observed in films subjected to a uniformly varying magnetic field, where the size of the free-volume holes initially decreased and subsequently increased over time. Conversely, a rotating magnetic field was found to enlarge the free-volume holes. Upon examining these phenomena, it appears that the magnetic field’s influence on crystalline orientation may be a contributing factor. The dielectric–frequency curve suggests that judicious magnetic field modulation can enhance dielectric properties. By examining the temperature dependence of the real part of the dielectric constant, the glass transition temperature (Tg) and thermal activation relaxation peak of the material were investigated. Through our analysis of the relationship between the imaginary part of the dielectric modulus and temperature, it was determined that the polymer films, after 3 min of treatment with a directionally constant magnetic field (M3), exhibited the most significant interfacial polarisation peak. Coupled with the D–E hysteresis loop, M3 demonstrated superior maximum polarisation. This indicates that magnetic field regulation is advantageous for dielectric polarisation and relatively reduces the remaining polarisation. Consequently, this can prevent thermal breakdown due to Joule heating from residual polarisation during the film’s charge–discharge process, as evidenced by the breakdown performance curve.

M3 exhibits the smallest free-volume pore size at 2.91 Å, the highest γ-phases content at 54.8%, the smallest grain size at 68 Å, the largest potential shift at 10.64, and a remarkably high discharge energy density of 12.68 J/cm^3^, representing a 200% enhancement compared to pure PVDF. Despite the fact that some samples possess superior dielectric properties to M3, it is evident that energy storage performance does not solely rely on the decoupling modulation of dielectric properties. Instead, it is influenced by a combination of factors, including free-volume pore size and γ-phase contents. For the highly polar polymer PVDF, the impact of free-volume pore size on its dielectric properties is quite intricate and is influenced by various elements such as phase structure, the dipole moment of polar groups, and dipole density, aligning with the findings reported in the literature [21,22,32]. In comparison to alternative approaches for boosting the dielectric properties of polymers, this method significantly reduces the preparation time and cost associated with dielectric energy storage materials. Most importantly, it offers a viable strategy for the research and development of innovative materials, which is a contribution of considerable significance to the field.

## Figures and Tables

**Table 1:** Crystal surface index, half-height width, and grain size of samples obtained by Jade 6.5 software.

Sample	hkl	FWHM	D/Å (A = 0.1 nm)
RM1	110	0.459	180
RM3	110	0.610	134
RM5	110	0.375	223
M1	110	0.393	213
M3	110	1.196	68
M5	110	0.438	189
Non	110	1.024	79

## Data Availability

All relevant data are within the manuscript.
